# Aryl hydrocarbon receptor activation increases survival in polymicrobial sepsis

**DOI:** 10.1186/cc14039

**Published:** 2014-12-03

**Authors:** A de Freitas, P Barbim Donate, F Vargas e Silva Castanheira, V Borges, D Carvalho Nascimento, J Talbot, JC Alves-Filho, F de Queiróz Cunha

**Affiliations:** 1 Department of Pharmacology, Faculty of Medicine of Ribeirão Preto, University of São Paulo, Ribeirão Preto, Brazil

## Introduction

Sepsis is a systemic inflammatory response resulting from the inability of the host to restrict the infection locally. Studies realized in our laboratory demonstrated that the high mortality observed in severe sepsis (S-CLP) correlates with the failure of the neutrophil migration to infectious focus and infection dissemination. However, animals subjected to a model of local infection (NS-CLP) present an efficient neutrophil recruitment that constrains the spreading of infection. In this context, we demonstrated a direct action of IL-17 mediating the neutrophil recruitment [1]. Recently, it was demonstrated that aryl hydrocarbon receptor (AhR) activation has a role in immune response. The AhR is expressed by Th17 cells and also by innate immune system cells and is important for their effectors functions, including IL-17 and IL-22 production [2]. However, the function of the AhR in sepsis remains uncertain. Herein, we investigate the role of AhR in polymicrobial sepsis induced by cecal ligation and puncture (CLP).

## Methods

C57BL/6 mice were subjected to NS-CLP or S-CLP sepsis. The protein expressions of AhR, CYP1A1 (indicator of AhR activation) and AhRR (AhR repressor) were determined in the spleen, liver and lung by western blot, 18 hours after sepsis. AhR and CYP1A1 were also evaluated, 6 hours after surgery, by RT-PCR in peripheral blood mononuclear cells (PBMC). Mice were pretreated subcutaneously with 30 μg/kg FICZ, high-affinity AhR agonist, 12 hours and 1 hour before the induction of moderate sepsis (M-CLP). Intraperitoneal neutrophil migration, bacteremia, kidney function and AhR activation were determined 6 hours after surgery. The survival rate was assessed every 12 hours up to 120 hours.

## Results

We observed reduced expression of AhR, CYP1A1 and increased AhRR expression in all analyzed organs of the S-CLP group compared with the NS-CLP group. Moreover, AhR and CYP1A1 were not detected in PBMC after S-CLP. Our results also demonstrated that activation of Ahr, through FICZ pretreatment, increased the neutrophil recruitment to the peritoneal cavity of mice subjected to M-CLP. Consequently, these animals presented reduced bacteremia and kidney injury, resulting in increased survival rate.

## Conclusion

These data suggest that during severe infection the AhR expression and activity is reduced and can contribute to the mortality observed in this process.
